# Editorial: Bone health and development in children and adolescents, volume II

**DOI:** 10.3389/fendo.2025.1720995

**Published:** 2025-10-20

**Authors:** Fátima Baptista, Federico Baronio

**Affiliations:** ^1^ CIPER, Faculdade de Motricidade Humana, Universidade de Lisboa, Lisbon, Portugal; ^2^ Pediatric Unit, Istituto di Ricovero e Cura a Carattere Scientifico (IRCCS) Azienda Ospedaliero-Universitaria di Bologna, Bologna, Italy

**Keywords:** mechanical loading, metabolic regulation, cardiometabolic index, bone mineral density (BMD), extracellular vesicles, cerebral palsy, pediatric densitometry, pycnodysostosis

Bone health and development in children and adolescents are key factors for lifelong skeletal strength. During adolescence, about half of an adult’s bone mineral content is accumulated, with over 80% of peak bone mass usually reached by age 18. This phase is vital not only for maintaining a healthy diet and establishing healthy eating habits but also for engaging in sufficient physical activity. Mechanical loading influences bone size and structure, while metabolic and hormonal factors control accrual and remodeling. Despite significant progress, gaps remain in understanding how modifiable and inherent factors interact *in vivo* and how to best evaluate bone health in a rapidly changing skeleton. This Research Topic compiles studies exploring the mechanical, metabolic, and psychosocial influences, as well as clinical conditions and diagnostic advancements, offering a comprehensive view of bone development during growth.

In this context, Erlandson et al. followed children engaged in recreational (non-competitive) gymnastics and observed sustained benefits at the distal radius, sites that are typically less exposed to habitual loading. Participants accumulated greater bone area, mineral content, and estimated strength at the wrist, with no apparent differences at the radial shaft or tibia. The pattern suggests modeling responses driven primarily by bone size rather than density, a distinction that matters for fracture resistance at a site where injuries peak during growth. These findings underscore the value of diverse, upper−limb loading in childhood, when rapid linear growth precedes mineral accrual and fragility transiently increases.

By contrast, Agostinete et al. examined adolescent competitive swimmers and found lower areal bone mineral density (aBMD) at the lower limbs and whole body compared with non−athletes, but significantly higher aBMD at the upper limbs. This regional pattern underscores the osteogenic role of muscular contractions in the arms, which experience repetitive loading despite the hypo-gravitational nature of swimming. Training load, defined as session rating of perceived exertion multiplied by training volume, was negatively associated with aBMD; however, inflammatory markers (IL-6, CRP) did not mediate this relationship. Swimmers presented with higher levels of calcium, vitamin D, and IGF-1, but were leaner and slightly shorter than the control group. Because DXA−derived aBMD is size−dependent, the absence of explicit height adjustment warrants caution when interpreting group differences. It provides a natural bridge to a dedicated study on pediatric densitometry.

Magallares et al. directly addressed this challenge by testing whether the height-for-age Z-score (HAZ) adjustment, following the approach of Zemel et al. (1), should be applied routinely in pediatric DXA interpretation. In 103 children and adolescents, discrepancies of up to 7% emerged between unadjusted and HAZ−adjusted Z−scores at the lumbar spine and whole body. Adjustment reduced misclassification at both ends of the height spectrum, correcting the underestimation of bone density in shorter individuals and the overestimation in taller ones, where bone size may confound aBMD. The study strengthens the case for systematic size correction in pediatric densitometry to improve diagnostic accuracy and comparability across cohorts.

Hodgson et al. used peripheral quantitative computed tomography to characterize tibial structure in children and adolescents with cerebral palsy and other neuromotor impairments. They reported markedly smaller and thinner long bones, with Z−scores for bone and muscle parameters ranging from -2.7 to -1.1. Deficits were greatest in those with poorer motor function. They reflected reduced periosteal expansion and cortical thickness rather than impaired cortical mineralization, an imprint consistent with reduced weight−bearing and attenuated muscular loading. These data reinforce the fundamentally mechanical nature of bone deficits in this population and support the use of targeted, weight-bearing, and muscle-strengthening strategies to reduce fracture risk.

Turning to metabolism, Liu et al. analyzed NHANES data from 1,514 adolescents aged 12–19 years. They observed an inverse association between the cardiometabolic index (a composite of triglycerides, HDL−cholesterol, and waist−to−height ratio) and BMD at the lumbar spine and femoral neck, but not at other proximal femur sites. These predominantly trabecular regions could be more metabolically sensitive to systemic dysregulation of lipid and glucose pathways. Notably, associations persisted after adjustment for BMI and physical activity, suggesting that cardiometabolic risk is related to impaired bone accrual in adolescence, particularly in individuals with central adiposity and an unfavorable lipid profile.

He et al. expanded the scope to include psychosocial factors by studying extracellular vesicle (EV) microRNAs (miRNAs) in participants with a history of early-life stress treated at a psychosomatic clinic. Twenty-two EV-miRNAs were differentially expressed compared to controls, with several associated with bone formation (P1NP), resorption (CTx), or turnover (osteocalcin). Notably, miR-26b-5p and miR-421, both involved in osteogenic differentiation but playing opposite roles —miR-26b-5p promotes and miR-421 inhibits osteogenesis —were linked to bone turnover markers in adjusted models. These results suggest that early psychosocial stress may influence the balance between bone formation and resorption through epigenetic regulation mediated by extracellular vesicle microRNAs, a hypothesis that calls for further mechanistic investigation.

Together, these studies illustrate a complex landscape where bone health during growth depends on the interaction of mechanical loading, metabolic regulation, and psychosocial factors. This physiological framework aids in understanding clinical cases where development is hindered by chronic disease, neuromotor impairment, or genetic predisposition, conditions in which the same biological mechanisms that support normal adaptation, when disrupted, can cause skeletal fragility.

Within clinical contexts, Alsaghier et al. reported a series of eight Saudi patients with pycnodysostosis due to a shared CTSK mutation (NM_000396.3(CTSK):c.244−29A>G) - seven of eight experienced fractures, pseudotumor cerebri, or hypophosphatemia. Response to growth hormone therapy was variable, underscoring the need for individualized care. The authors argue for early genetic screening and a multidisciplinary approach to optimize outcomes.

Cao et al. synthesized evidence on Modic changes in the lumbar spine across 25 studies, estimating an overall occurrence of ~35%. Age, disc degeneration, endplate changes, spondylolisthesis, reduced anterior lumbar lordosis, and a history of physical labor were significant risk factors, whereas sex, BMI, and smoking showed no consistent associations. These insights provide a framework for earlier recognition and targeted management of spine pathology.

Chutani et al. investigated an enriched botanical extract, Fortified Withaferin A (FWA), as a potential enhancer of endochondral bone repair. In rodents, the healing time was reduced from 23–24 days to approximately 12 days, with FWA downregulating osteoclast-related genes, promoting anabolic responses, and reducing inflammation, effects that exceeded those of parathyroid hormone in regulating osteoclasts. While further translational steps are needed, the data support assessing FWA as a cost-effective adjunct in the healing of delayed or osteoporotic fractures.

Cun et al. evaluated the systemic inflammatory response index (SIRI) in 8–19-year-olds and, contrary to many adult reports, observed positive associations with BMD in the pelvis, trunk, and whole body. The authors speculate that moderate inflammation may accompany remodeling during growth, whereas excessive inflammation may be harmful. Sex, age, and BMI influenced these associations, with more favorable outcomes in males and less so in those with obesity, highlighting SIRI as a potential biomarker for early, personalized risk assessment.

In a heterogeneous clinical cohort of children and adolescents referred for bone health assessment, Magallares et al. reported that over 96% of the 103 patients who already exhibited a risk factor showed two or more, which were often unnoticed. The prevalence of low bone mass for chronological age was 10.5%, and childhood osteoporosis was 4.8%. Male sex was positively associated with vertebral BMD, while a sedentary lifestyle and previous fractures were negatively correlated with whole-body and head BMD. Physical inactivity proved to be the most modifiable risk factor, supporting the routine use of DXA morphometry and targeted interventions.

Zhang et al. focused on transfusion-dependent β-thalassemia and showed that the initially high prevalence of low bone mass (31.6%) decreased to 15.8% after height-adjusted BMD correction. Increasing age was a consistent risk factor; in unadjusted models, IGF-1 levels below -2 SD and hypogonadism were prominent risks, whereas, after adjustment, normal BMI and higher albumin levels were found to be protective. The study highlights the clinical utility of height−adjusted BMD, alongside assessment of the growth hormone axis, gonadal function, and nutritional status.

Wang et al. combined a challenging case with a systematic review to clarify surgical timing in Blount’s disease. For children with onset before four years, conservative management followed by timely osteotomy achieves recovery rates of up to 80%. In those over ten years, growth potential assessment guides the strategy: hemiepiphysiodesis is suitable for patients with at least two years of remaining growth, while osteotomy is preferred otherwise. Delayed or inappropriate interventions increase the risk of recurrence and burden, emphasizing the importance of personalized planning.

The collective evidence presented in this Research Topic emphasizes the connection between physiological adaptation and clinical vulnerability. Advances in understanding how mechanical loading, metabolic status, and mental health interact with bone modeling are enhanced by improved diagnostic tools, such as size-adjusted pediatric DXA. Protecting skeletal health during growth, therefore, requires combining these perspectives to encourage diverse physical activity, balanced nutrition, metabolic fitness, and psychosocial well-being, ensuring that growth benefits translate into lifelong skeletal resilience.
